# Stock profiling using time–frequency-varying systematic risk measure

**DOI:** 10.1186/s40854-023-00457-7

**Published:** 2023-02-14

**Authors:** Roman Mestre

**Affiliations:** Montpellier Research in Economics, University of Montpellier, Avenue Raymond Dugrand, 34960 Montpellier, Cedex 2, France

**Keywords:** Maximal overlap discrete wavelets transform, Time, Frequency-varying beta, Time, Frequency rolling window, Risk-profile, Systematic risk, G12, G11, C40, C32, C58, G40

## Abstract

This study proposes a wavelets approach to estimating time–frequency-varying betas in the capital asset pricing model (CAPM) framework. The dynamic of systematic risk across time and frequency is analyzed to investigate stock risk-profile robustness. Furthermore, we emphasize the effect of an investor’s investment horizon on the robustness of portfolio characteristics. We use a daily panel of French stocks from 2012 to 2022. Results show that varying systematic risk varies in time and frequency, and that its short and long-run evolutions differ. We observe differences in short and long dynamics, indicating that a stock’s betas differently fluctuate to early announcements or signs of events. However, short-run and long-run betas exhibit similar dynamics during persistent shocks. Betas are more volatile during times of crisis, resulting in greater or lesser robustness of risk profiles. Significant differences exist in short-run and long-run risk profiles, implying a different asset allocation. We conclude that the standard CAPM assumes short-run investment. Then, investors should consider time–frequency CAPM to perform systematic risk analysis and portfolio allocation.

## Introduction

The main objective of a portfolio manager or analyst is to assess an investment’s risk level (in an equities portfolio). The most well-known benchmark for this purpose is the capital asset pricing model (CAPM) (Sharpe 1964), based on Markowitz’s mean–variance approach. Professionals and researchers have long been interested in improving the CAPM model. Numerous quantitative approaches and methods have been developed to solve some limitations of the CAPM or Markowitz’s portfolio optimization frameworks.

Moreover, a large portion of the literature focuses on improving risk estimation in portfolio optimization. Financial network models enable a better understanding of the complex interactions between the various assets in a portfolio (Hautsch et al. 2015; Baitinger et al. 2017). Thus, Clemente et al. (2021) modified the model’s optimization program with financial network models. Yang et al. (2021) subsequently improved on this approach with a multiobjective model including idiosyncratic variance. Moreover, Yu and Chang (2020) employed network models to account for the effects of macroeconomic variables. They found that these approaches provide more diverse portfolios with better performance than standard methods. Following the development of Fintech, professionals are increasingly using quantitative approaches to assist financial and banking industries in lowering the costs of technology applications while increasing the quality of their services (Kou et al. 2021a, b). According to Kou et al. (2021a, b), the banking sector is motivated to invest in Fintech to gain a competitive advantage and payment and money transfer systems. Furthermore, as part of Fintech, many new mathematical and statistical approaches are being developed to improve financial risk analysis. According to Kou et al. (2021a, b), a two-stage multiobjective feature selection model is used to predict SME bankruptcy and improve firms’ performance classification. However, Fintech new approaches can also be used to improve portfolio allocation using automatic algorithms, as emphasized by Guidici et al. (2022). They associated the financial network with the random matrix theory (RMT) in their studies to differentiate the nature of a correlation based on a systematic or noise component. Many authors, including León et al. (2017), Raffinot (2017), and Ren et al. (2017), have demonstrated the value of using RMT to improve portfolio performance.

Another literature section focuses on using wavelets’ time–frequency analysis to measure relationships between financial assets across various investment horizons (or macroeconomics cycles). The wavelets extract frequency components from time series while preserving their time information. Wavelets are then an extension of the spectral and cross-spectral analysis. The wavelets approach, which was originally developed for signal processing, is well suited to decomposing a time series into time–frequency space. In finance and economics, frequency is related to the period of cycles that comprise the original time series and is then applied to the investor’s investment horizon. Depending on the frequency grid, two wavelet transforms were developed: the maximal overlap discrete wavelets transform (MODWT) and the continuous wavelets transform (CWT). The primary distinction is the frequency grid used to select the accuracy of the results in the time and frequency domains. The MODWT provides frequency bands using a dyadic scale, whereas the CWT is finer and more accurate in extracting each frequency. Consequently, the computational time and effort differ in practice. When compared to the CWT approach, MODWT requires less computational effort.

The studies of Gençay et al. (2003, 2005) were the first to use discrete wavelets decomposition in finance. They used a time–frequency least squares regression to estimate the beta parameter of the CAPM market line and to analyze the frequency-varying systematic risk and the frequency risk profiles of the stocks. They emphasize the wavelets’ interest in assessing the frequency dynamics of systematic risk. Other authors use wavelets in finance as a result of their research. Several studies have focused on its use in finance to model interdependencies and relationships between commodities, stock indexes, and other financial assets such as cryptocurrencies (Rua and Nunes 2012; Vacha and Barunik 2013; Aguiar-Conraria and Soares 2014; Bekiros and Marcellino 2014; Bekiros et al. 2016; Kahraman and Unal 2019). Recently, partial continuous wavelet approaches have been developed to examine better relationships between various financial asset classes, such as equities or cryptocurrencies (Athari and Hung 2022), Islamic equities (Al-Yahyaee et al. 2020), or metals and energy future prices (Michis 2022). These studies discover that investment horizons (frequencies) influence risk’s diffusion. As a result, the characteristics of the assets and their degree of correlation are related to different time–frequency schemes describing risk contagion mechanisms.

As Gençay et al. (2003, 2005) demonstrated, using wavelets and a time–frequency analysis allows us to differentiate the beta parameter of the CAPM based on different investment horizons. The beta parameter of the market’s line is commonly used to establish an equity risk-profile. A beta of 1 indicates that the stock follows market movement in the same proportions that a beta greater (respectively lesser) than 1 indicates that the stock amplified (respectively attenuated) market fluctuation. Their results imply that the risk-profile (of a portfolio or a stock) is frequency dependent because the beta is frequency-varying. Mestre and Terraza (2018), McNevin and Nix (2018), Shah et al. (2018), and Sakemoto (2020) all confirmed that systematic risk is conditional on an investment horizon (frequencies). These studies also discovered that frequency dynamics differ across industries. These results are also observed and confirmed for CAPM extensions, such as Sakemoto’s (2020) intertemporal CAPM and Mestre’s (2021) arbitrage pricing theory (APT) and Fama–French models. Alexandridis and Hasan (2020) used wavelets to investigate the effect of the Global Financial Crisis on systematic risk. They found that the beta rises during the crisis period compared to the pre-crisis period, particularly at lower frequencies.

The CAPM’s hypothesis of homogeneity of the agents’ behavior can then be relaxed using wavelets. In this case, we assume that agents are distinguished by their trading behavior related to a specific investment horizon (short-run investors vs. long-run investors). We suppose that agents’ investment strategies should include a better measure of the market risk that is more in line with their trading behaviors. As a result, we propose comparing short and long-run estimations of the CAPM market’s line to discuss the robustness of the stock’s risk-profile across frequency.

Previous research has shown that systematic risk (the beta) varies with frequency. However, the authors assume that it is time-invariant. This is a well-known issue in the literature. Numerous studies on the CAPM in the literature highlight the instability of the beta parameter over time (Black et al. 1972; Fama and McBeth 1973; Fabozzi and Francis 1978). To overcome these constraints, scholars have used various methods, such as the GARCH process, Kalman–Bucy filter, or rolling window, to perform forward regression (Faff et al. 1992, Faff and Brooks 1998; Brooks et al. 1992, 1998; Groenewold and Fraser 1997). The time-varying systematic risk is an important extension of the CAPM to monitor the risk-profile time-robustness for various market conditions (e.g., expansion, turmoil, and crisis), especially with the recent COVID-19 pandemic and Ukrainian conflict affecting market conditions. Lopez et al. (2022) demonstrated that static beta estimation is irrelevant in COVID-19 because time-varying parameters are more appropriate. Similarly, Jain (2022) emphasized the value of time-varying betas. She observed increasing betas in the Indian stock market during the first COVID-19 wave but not during the second wave. She also claimed that beta dynamics are affected by firm sectors.

Many studies in the literature, such as Zhang et al. (2020), He et al. (2020), and Hui and Chan (2022), highlighted the increasing risk during the COVID-19 crisis confirming Alexandridis and Hassan (2020)’s results for the Global Financial Crisis. However, they did not include wavelet decomposition. The main goal of the preset study is to highlight the role of investment horizons on systematic risk in different market conditions. Following the research of Mestre and Terraza (2019) and McNevin and Nix (2018), we combine time-varying estimation and frequency decomposition. They proposed a rolling time–frequency estimation of the market line and developed the concept of time–frequency-varying beta parameters. The evolution of time–frequency-varying betas can highlight various systematic risk patterns in the short and long-run. They demonstrated, among other things, that the time dynamics of systematic risk differ significantly, depending on the frequencies used. Therefore, we assume that various time–frequency patterns are influenced by agents’ perceptions of market conditions related to risky (or not) events. Furthermore, time–frequency-varying systematic risk assume that portfolio features (in the short or long-run) can change compared with a standard approach. The robustness of stock/portfolio risk profiles during expansion and turmoil periods can then be investigated. Thus, a risk-profile can be considered robust or not based on the volatility of the beta over time and frequency. Thus, analyzing systematic risk in the time–frequency space can induce adjustments in portfolio allocation based on the time and frequency characteristics of stocks.

This study uses the time–frequency rolling window developed by Mestre and Terraza (2019) to analyze the time–frequency robustness of the initial risk-profile. We use the daily returns of selected stocks and the Eurostoxx index from 2012 to June 2022. Moreover, we provide a method for promptly assessing the time–frequency characteristics of stocks or portfolios.

We also analyze the short-run and long-run patterns of systematic risk. We confirm the frequency differentiation of systematic risks observed in the literature and find that risk dynamics differ as well. The short-run patterns of time–frequency betas closely resemble the standard CAPM time-varying estimation approach, whereas the long-run pattern differs. We also highlight that the static CAPM beta’s initial value influences the stock risk-profile’s time–frequency robustness. The closer the beta is to 1, the less robust the risk-profile. These results are observed during periods of expansion and turmoil. However, during crisis, beta volatility is higher, resulting in drastic changes in stock and portfolio risk profiles. The different reactions of short-run and long-run betas to the early stages of the COVID-19 and Ukrainian conflict suggest that agents perceive these events differently depending on their investment horizon.

The remainder of this paper is structured as follows. Section 2 presents the theoretical aspects of time–frequency approach. Section 3 estimates the time–frequency-varying betas using the standards rolling approach and the time–frequency-varying rolling window. We use a standard approach to compare short and long-run time–frequency dynamics. We also created three portfolios with distinct features and present our methodology for evaluating the time–frequency robustness of their respective risk profiles. Section 4 examines the time–frequency robustness of these portfolios’ risk profiles during the COVID-19 and Ukraine war periods (2020–2022). Finally, Sect. 5 concludes the paper.

## Theoretical aspects of wavelets time–frequency analysis in the CAPM framework

This section discusses the various types of wavelet decomposition and the approach used to perform time–frequency-varying estimations. The mathematical theory is presented using Mallat’s (2009a) notation. The frequency decomposition of time series results in the loss of time representation of a time series’ frequency components. The wavelets decompositions are then an extension of the spectral analysis to overcome this limitation (Meyer et al. 1986; Grossmann and Morlet 1984; Meyer 1990; Mallat 1989, 2009a, b; Daubechies 1992). The decomposition of a wavelet is then based on a wavelet-mother, $\Psi \left(\mathrm{t}\right),$ which uses a filter basis of a time series $x(t)$ to extract frequency components while maintaining a time representation of them. The wavelet-mother should respect a admissibility conditions to preserver the energy/variance of the initial time series $x\left(t\right)$ (Grossmann and Morler 1984).

Consequently, the wavelet $\Psi \left(\mathrm{t}\right)$ is a zero-mean and normalized function written as follows:

1 $$\int_{ - \infty }^{ + \infty } {\psi \left( t \right)} dt = 0\;\; {\text{and }}\int_{ - \infty }^{ + \infty } {\left| {\psi \left( t \right)^{2} } \right|} dt = 1$$

The wavelets $\Psi \left(\mathrm{t}\right)$ is shifted by a parameter $\uptau$ and scaled by the parameter s to generate the wavelets-daughters ${\Psi }_{\uptau ,\mathrm{s}}\left(\mathrm{t}\right)$ representing the wavelets-mother in different subspaces determined by varying s and $\uptau$:

2 $${\Psi }_{\uptau ,\mathrm{s}}\left(\mathrm{t}\right)=\frac{1}{\sqrt{\mathrm{s}}}\Psi \left(\frac{\mathrm{t}-\uptau }{\mathrm{s}}\right)$$

Consequently, by considering all the wavelets-daughters across s and $\uptau$, we project the chronic $x\left(t\right)$ into each subspace ${\Psi }_{\uptau ,\mathrm{s}}\left(\mathrm{t}\right)$ to have the wavelets coefficients $W\left(s,\tau \right)$ at each scale s and across time:

3 $$W\left(s,\tau \right)={\int }_{-\infty }^{+\infty }x\left(t\right){ \frac{1}{\sqrt{s}}\psi }^{*}\left(\frac{t-\tau }{s}\right)dt$$

$${\psi }^{*} \, is \, the\, complex \,conjugate\, of \, \psi$$

The wavelet coefficients represent variations of the original series at frequency scale s and time scale t. As a result, the CWT generates a sub-chronic with the same length N as the initial time series at each frequency scale. We can reconstruct the initial chronic $x(t)$ by the reverser process known as the inverse continuous wavelets transform (ICWT):

4 $$x\left( t \right) = { }\frac{1}{{C_{\psi } }}\int_{ - \infty }^{ + \infty } {\int_{ - \infty }^{ + \infty } {\psi_{{\uptau ,{\text{s}}}} \left( t \right)} } \;W\left( {s,\tau } \right)\frac{{{\text{d}}\uptau {\text{ds}}}}{{{\text{s}}^{2} }}$$

Equation (4) highlights the admissibility conditions ${C}_{\psi }$ of the wavelet-mother (Calderon 1964; Daubechies 1992):

5 $$C_{\psi } = \int_{0}^{ + \infty } {\frac{{\left| {\widehat{\Psi }\left( f \right){ }} \right|^{2} }}{f}} \;df < + \infty$$

where $f{\text{ is the frequency and }}\hat{\Psi }\left( f \right)$ is the Fourier’s transform of wavelets-mother.

Equation (5) is respected if the wavelets-mother satisfies Eq. (1). In this paper, we use the complex Morlet wavelets ${\psi }_{M}\left(t\right)$ as wavelet-mother because it provides a good balance in the time and frequency representation.

6 $${\psi }_{M}\left(t\right)= {\pi }^{-1/4}{e}^{i{f}_{0}t}{e}^{(-\frac{{t}^{2}}{2})}$$

With ${i}^{2}=-1$ and ${f}_{0}$, the non-dimensional frequency is equal to 6 to satisfy the condition on ${C}_{\psi }$.

In practice, frequency sampling is used to empirically implement the CWT (Lau and Weng 1995; Torrence and Compo 1989), specifying the optimal level of decomposition, noted J, and the related set of frequency scale noted,${s}_{j}$, depending on the time step, ${\delta }_{t},$ of the time series and a frequency step, $\delta_{s}$:

7 $$\begin{array}{*{20}l} {s_{j} = { }s_{0} .{ }2^{{j\delta_{j} }} } \hfill \\ {\forall {\text{ j}} = 0, \ldots .{\text{J and }}s_{0} = 2*\delta_{t} } \hfill \\ \end{array}$$

8 $$J = { }\frac{1}{{\delta_{j} }}{ }\left\lfloor {Log_{2} \left( {\frac{{N\delta_{t} }}{{s_{0} }}} \right)} \right\rfloor = \frac{1}{{\delta_{j} }}{ }\left\lfloor {Log_{2} \left( \frac{N}{2} \right)} \right\rfloor$$

In this framework, the degree of frequency resolution of the CWT can be modified by varying ${\delta }_{s}$ as ($\frac{1}{{\delta }_{j}}-1)$ intermediary frequencies will be caught between two dyadic scales. The value of ${\delta }_{s}$ is also related to the wavelets-mothers chose to perform the CWT.

The CWT is then relatively complex to implement, necessitating the development of discretized versions to ease practical use by reducing computational efforts. The discrete wavelet transform and, in particular, its improved version, the MODWT, are the most well-known. The MODWT is theoretically a simplification of the CWT because it employs a dyadic frequency scale grid and the cascade algorithm (Mallat 1989, 2009a, b) to recursively decompose the initial time series while retaining its variance. The outputs of the MODWT are the frequency bands instead of frequency. In this framework, the frequency bands regroup frequencies between each multiple of 2, until an optimal order of decomposition J is found. J is then the number of frequency bands resulting from a MODWT that can be used to describe the original series $x\left(t\right)$ without losing information.

9 $$J=\frac{Ln(N)}{Ln(2)}$$

The MODWT is based on the Mallat’s cascade algorithm, which uses low-pass and high-pass filters to successively decompose J time the time series. The wavelets-mother is a high-pass filter, and the wavelet-father $\phi \left(t\right)$ is a low-pass filter (also called scaling function). The previous ${\psi }_{\tau ,s}\left(t\right)$ and ${\phi }_{\tau ,s}\left(t\right)$ are the wavelet-mother and father shifted by $\tau$ and dilated by $s$.

Mallat’s algorithm is completely iterative because the previous filters are applied to the initial series and the output of the filtering J times while taking rescaling and subsampled coefficients into account at each step j (Gençay et al. 2003). In addition, the MODWT provides sub-chronic at each step j = 1,….J known as the detail coefficients ${D}_{j}$ or frequency bands (similar to the wavelets coefficient in the CWT case). The frequency bands describe the fluctuation on a range of frequencies related to the periods of these cycles (see Appendix 1). The high-frequency bands capture short-run fluctuations, whereas the low-frequency bands capture long-run fluctuations. Note that ${D}_{j}$ has the same length N as the initial series. The MODWT provides the related frequency bands ${D}_{J}$ and a rough approximation of the ${S}_{J}$ series for the final level J. This approximation is usually the series mean or a basic trend approximation. By adding the frequency bands, we can reconstruct the original series:

10 $${x}_{t}= {\mathrm{S}}_{\mathrm{J},\mathrm{x}}+ \sum_{\mathrm{j}=1}^{\mathrm{j}=\mathrm{J}}{\mathrm{D}}_{\mathrm{j},\mathrm{x}}$$

Because the MODWT provides frequency bands of length N, the wavelets variance of each band can be computed as follows:

11 $$V\left({\mathrm{D}}_{\mathrm{j},\mathrm{x}}\right)=\frac{1}{{ N}_{j}}\sum_{t}^{N-1}{\widetilde{{\varvec{d}}}}_{j,x,t}^{2}$$

where ${\widetilde{{\varvec{d}}}}_{j,\mathrm{x}, t}^{2}$ are the wavelets coefficients at the level j. ${N}_{j}$ is the number of coefficients non-affected by boundary (see Gençay et al. 2003).

In a multivariate framework, considering another time series ${y}_{t}$, we can compute the wavelets covariance between the frequency bands of level j of each series such as:

12 $$Cov\left({\mathrm{D}}_{\mathrm{j},\mathrm{ x}},{\mathrm{D}}_{\mathrm{j},\mathrm{y}}\right)=\frac{1}{{ N}_{j}}\sum_{t}^{N-1}{\widetilde{{\varvec{d}}}}_{j, x,t}{\widetilde{{\varvec{d}}}}_{j,y,t}$$

Consequently, for each frequency band, we can perform a time–frequency coefficient, ${\beta }_{j}$ as follows:

13 $${\beta }_{j}=\frac{Cov({\mathrm{D}}_{\mathrm{j},\mathrm{ x}},{\mathrm{D}}_{\mathrm{j},\mathrm{y}})}{V\left({\mathrm{D}}_{\mathrm{j},\mathrm{x}}\right)}=\frac{\frac{1}{{ N}_{j}}\sum_{t}^{N-1}{\widetilde{{\varvec{d}}}}_{j, x,t}^{2}{\widetilde{{\varvec{d}}}}_{j,y,t}^{2}}{\frac{1}{{ N}_{j}}\sum_{t}^{N-1}{\widetilde{{\varvec{d}}}}_{j,x,t}^{2}}$$

In our paper, we used the following market’s line equation of the basic Sharpe’s CAPM to establish a relationship between asset risk premium ${y}_{t}$ and market premium ${x}_{t}$.

14 $${y}_{t}=\alpha +{\beta .x}_{t}+{\varepsilon }_{t}$$

In Eq. (14), ${y}_{t} \, and \, {x}_{t}$ are stationary processes and ${\varepsilon }_{t}$ is a i.i.d(0,${\sigma }_{\varepsilon }$) process.

Equation (13) provides a constant coefficient for each frequency band based on the standard representation of the market’s line. However, a rolling time–frequency window can be used to estimate a time-varying coefficient on each band (Mestre and Terraza 2019; McNevin and Nix 2018). McNevin and Nix (2018) used an L-sized rolling window to estimate time-varying betas on each frequency band from a unique MODWT. This approach is applicable and intuitive for evaluating the time–frequency-varying beta parameter in the CAPM framework. However, it is based on a unique wavelet decomposition based on N points (the length of the initial series decomposed), whereas the estimation is realized into the window size (based on L points). As a result, the wavelet coefficients are not rescaled or subsampled because the algorithm used an N-based MODWT to perform these operations. Such operations are useful for satisfying decomposition properties, particularly variance preservation. Furthermore, as Mestre and Terraza (2019) demonstrated, some bias can affect parameter estimations, particularly in the long-run.

Mestre and Terraza (2019) proposed the time–frequency rolling window performing the MODWT inside the L-sized window. Thus, all rescaling and subsampling operations are compatible with estimating the beta parameter based on L points. Furthermore, the empirical application of this approach is tailored to the price discovery process because the window incorporates the new prices directly into the computation, whereas the intuitive approach necessitates redefining the basis points used to perform the MODWT. They compared these two approaches in their paper’s CAPM framework and concluded that the time–frequency rolling window provides more accurate estimators than the intuitive approach.

## Time–frequency-varying systematic risk and profiling of stocks and portfolios

The time–frequency rolling window is applied to French stocks listed on the CAC40 indexes for the daily period 2012–2019. We retain the Eurostoxx index as the Market index (factor) and the OAT 10-year rates as a risk-free asset. Then, we compute the log returns (first difference of natural logarithm of daily closed prices) to generate the market premium and equities premia variables of the market’s line. Using these variables, we estimate a static beta by ordinary least square (OLS) and rolling beta series, denoted ${\beta }_{r},$ using a 260-day rolling window (1 trading year). Investors can change the size of the window to suit their needs. The results will be qualitatively similar. The static betas are used as a benchmark to establish the initial risk-profile of each stock based on their respective values relative to 1 (greater, lower, equal to 1), whereas the ${\beta }_{r}$ is used to analyze the time dynamics of risk.

Furthermore, we employ the MODWT transformation in conjunction with a least-asymmetric Daubechies wavelet (La8) to estimate frequency betas parameters by OLS. The investment horizon associated with each frequency band is presented in Appendix 1. To simplify the analysis and interpretation of the results, we keep only the short-term band D1 and the long-term band D6, which correspond to investment horizons of 2 to 4 days and 3 to 6 months, respectively. The betas calculated for D1 and D6 are time-invariant and are used to establish the frequency risk profiles. Then, by comparing their respective values, we analyze the frequency robustness of the initial risk-profile. To understand the time dynamics of these parameters, we use Mestre and Terraza’s (2019) approach (with a 260-day time–frequency rolling window) to estimate the rolling time–frequency betas noted ${\beta }_{TF}$. We obtained a time series representing the beta for a particular frequency band.

Based on an analysis of the time–frequency dynamics of the betas, we propose the following methods for quantifying the robustness of the risk-profile over time and across frequencies.

To categorize each stock using standards CAPM and stand the initial risk-profile, we test the value of the static beta (equal, greater, or lesser than 1). We calculate the standard deviation of the rolling betas series to quantify the degree of betas volatility and then the time-robustness of the initial risk-profile. The rolling beta values are tested to 1 and compared to the initial risk-profile. We count the number of rolling betas greater, equal, or lesser than 1 and 0 (if required). As a result, when a large portion of the rolling betas match the initial risk-profile, the risk-profile is assumed to be robust over time. Conversely, when rolling betas fail to satisfy initial characteristics, the initial risk-profile is supposed to be non-robust.

We follow this methodology for ${\beta }_{r}$ and ${\beta }_{TF}$ on D1 and D6 frequency bands, and the results are recorded in Table 1. The first two columns in these tables present the OLS beta and its T-stat to test its equality to 1. The fifth column calculates the rolling beta series’ standard deviation, and the last columns show the percentage of rolling betas in each category (greater, lesser and equal to 1 and 0). The Sharpe and Treynor ratios are also assigned. However, in the frequency domain, they are closed to 0 because frequency bands are zero-mean variables. Tables 1 presents all the results.

**Table 1:** Stocks time–frequency risk characteristics

2012–2019	OLS beta	Tstat1	Sharpe	Treynor	SD	%Beta < 1	%Beta = 1	%Beta > 1	%Beta < 0	%Beta = 0
*a) For stocks with an OLS beta equal to 1*										
Airbus	0.97	1.08	− 54.91	− 0.97	0.17	33.01	24.34	42.64	0	0
Airbus D1	0.95	2.01	0.00	0.00	0.20	44.38	19.26	36.36	0	0
Airbus D6	1.24	8.70	− 0.07	0.00	0.63	38.40	2.15	55.68	2.57	1.20
LVMH	1.00	0.19	− 63.14	− 0.95	0.22	39.29	20.33	40.37	0	0
LVMH D1	1.03	1.43	0.04	0.00	0.20	40.01	20.33	39.65	0	0
LVMH D6	1.06	2.35	− 0.06	0.00	0.35	23.09	6.58	70.16	0	0.18
*b) For stocks with an OLS beta lesser than 1*										
Hermès	0.57	18.76	− 77.20	− 1.70	0.18	99.46	0.54	0	0	0
Hermès D1	0.54	19.69	0.01	0.00	0.20	99.88	0.12	0	0	0
Hermès D6	0.45	18.96	− 0.05	0.00	0.58	58.07	7.18	17.70	10.23	6.82
Ricard	0.59	20.56	− 85.14	− 1.68	0.11	100	0	0	0	0
Ricard D1	0.60	19.95	− 0.01	0.00	0.14	100	0	0	0	0
Ricard D6	0.67	15.67	− 0.14	0.00	0.36	74.82	9.99	8.25	2.99	3.95
Dassault	0.60	16.00	− 70.02	− 1.57	0.26	84.81	1.91	13.28	0	0
Dassault D1	0.60	16.01	− 0.01	0.00	0.30	83.85	2.51	13.64	0	0
Dassault D6	0.52	19.61	− 0.01	0.00	0.66	56.16	3.95	8.67	27.03	4.19
Thales	0.61	16.49	− 72.43	− 1.56	0.11	100	0	0	0	0
Thales D1	0.60	17.55	− 0.01	0.00	0.13	100	0	0	0	0
Thales D6	0.51	18.75	− 0.17	0.00	0.38	72.25	6.22	13.82	5.98	1.73
Publicis	0.65	13.04	− 68.46	− 1.57	0.08	100	0	0	0	0
Publicis D1	0.59	15.01	0.00	0.00	0.07	100	0	0	0	0
Publicis D6	0.66	14.20	0.15	0.00	0.60	56.40	2.87	26.38	11.72	2.63
Téléperformance	0.65	12.40	− 58.83	− 1.37	0.18	91.69	0.84	7.48	0	0
Téléperformance D1	0.60	14.28	0.01	0.00	0.21	91.87	0.66	7.48	0	0
Téléperformance D6	0.40	22.92	.0.00	0.00	0.48	67.17	2.39	12.14	15.31	2.99
Danone	0.68	17.13	− 86.03	− 1.48	0.10	98.50	1.50	0	0	0
Danone D1	0.72	15.43	0.04	0.00	0.09	99.94	0.06	0	0	0
Danone D6	0.67	18.92	− 0.09	0.00	0.29	84.75	6.76	4.49	1.38	2.63
Alstom	0.72	9.46	− 61.62	− 1.37	0.16	92.46	7.06	0.48	0	0
Alstom D1	0.71	10.53	0.03	0.00	0.14	94.38	4.31	1.32	0	0
Alstom D6	0.70	7.82	− 0.09	0.00	0.63	63.10	5.80	30.50	0.36	0.24
Eurofins	0.74	7.36	− 50.54	− 1.26	0.25	83.97	1.20	14.83	0	0
Eurofins D1	0.69	9.05	− 0.05	0.00	0.28	86.12	6.28	7.60	0	0
Eurofins D6	0.66	11.38	− 0.01	0.00	0.59	50	7.30	27.69	11.78	3.23
Veolia	0.75	9.51	− 64.21	− 1.31	0.12	98.80	1.20	0	0	0
Veolia D1	0.66	12.48	0.00	0.00	0.14	100	0	0	0	0
Veolia D6	0.75	8.14	− 0.07	0.00	0.47	60.94	8.25	27.99	2.39	0.42
L’oreal	0.78	11.69	− 78.25	− 1.24	0.12	98.03	1.97	0	0	0
L’oreal D1	0.82	9.58	0.06	0.00	0.12	89.17	10.77	0.06	0	0
L’oreal D6	0.71	15.97	− 0.13	0.00	0.24	86.24	10.77	2.99	0	0
Essilor	0.78	9.75	− 72.47	− 1.26	0.11	96.35	3.65	0	0	0
Essilor D1	0.80	9.19	− 0.02	0.00	0.13	94.80	5.20	0	0	0
Essilor D6	0.62	15.97	0.17	0.00	0.46	72.91	3.65	13.10	9.39	0.96
Vivendi	0.79	9.08	− 70.06	− 1.25	0.14	84.27	7.95	7.78	0	0
Vivendi D1	0.79	8.76	0.02	0.00	0.16	79.19	9.99	10.83	0	0
Vivendi D6	0.68	12.57	− 0.19	0.00	0.40	67.94	4.96	26.14	0.42	0.54
Legrand	0.80	10.63	− 76.41	− 1.21	0.09	94.98	5.02	0	0	0
Legrand D1	0.76	12.47	0.01	0.00	0.10	96.23	3.77	0	0	0
Legrand D6	0.92	4.27	− 0.11	0.00	0.28	57.18	9.21	33.61	0	0
Safran	0.83	7.30	− 66.14	− 1.12	0.17	72.07	3.65	24.28	0	0
Safran D1	0.76	10.52	− 0.01	0.00	0.17	73.03	20.33	6.64	0	0
Safran D6	0.76	12.17	− 0.02	0.00	0.40	48.80	9.87	39.77	0.24	1.32
Air liquide	0.85	9.56	− 82.79	− 1.16	0.12	77.15	18.00	4.84	0	0
Air liquide D1	0.88	7.56	0.05	0.00	0.10	69.44	30.56	0	0	0
Air liquide D6	0.96	2.47	− 0.09	0.00	0.38	53.17	7.83	38.58	0	0.42
Sanofi	0.85	7.04	− 73.09	− 1.17	0.13	77.09	22.19	0.72	0	0
Sanofi D1	0.92	4.06	0.02	0.00	0.16	68.24	18.00	13.76	0	0
Sanofi D6	0.66	13.91	− 0.13	0.00	0.42	68.60	4.72	16.69	5.92	4.07
Michelin	0.86	5.81	− 64.59	− 1.15	0.13	81.34	5.14	13.52	0	0
Michelin D1	0.81	7.81	0.02	0.00	0.12	76.02	22.61	1.38	0	0
Michelin D6	0.88	5.45	− 0.03	0.00	0.49	48.74	9.99	37.98	1.38	1.91
Engie	0.89	4.93	− 71.64	− 1.15	0.06	80.44	19.56	0	0	0
Engie D1	0.84	7.18	0.06	0.00	0.11	83.97	16.03	0	0	0
Engie D6	0.90	4.22	0.14	0.00	0.40	58.85	8.01	33.13	0	0
Vinci	0.90	5.53	− 73.45	− 1.07	0.13	55.08	37.86	7.06	0	0
Vinci D1	0.89	5.92	0.07	0.00	0.14	59.15	31.16	9.69	0	0
Vinci D6	0.60	22.79	− 0.13	0.00	0.33	77.99	9.09	12.86	0	0.06
Bouygues	0.90	3.39	− 58.32	− 1.09	0.14	48.86	24.40	26.73	0	0
Bouygues D1	0.88	4.09	0.02	0.00	0.20	45.93	10.41	43.66	0	0
Bouygues D6	0.75	8.48	− 0.12	0.00	0.56	55.68	8.97	35.35	0	0
Orange	0.92	3.50	− 66.95	− 1.09	0.20	54.01	24.76	21.23	0	0
Orange D1	0.94	2.77	0.00	0.00	0.21	57.24	14.83	27.93	0	0
Orange D6	0.87	4.24	− 0.13	0.00	0.67	64.11	3.65	24.28	5.80	2.15
Kering	0.92	2.74	− 55.55	− 1.01	0.27	58.79	11.60	29.61	0	0
Kering D1	0.93	2.54	0.00	0.00	0.27	44.62	20.39	34.99	0	0
Kering D6	1.08	2.89	− 0.06	0.00	0.54	35.29	7.24	55.80	0.72	0.96
Capgemini	0.92	2.95	− 59.42	− 1.03	0.16	77.27	6.52	16.21	0	0
Capgemini D1	0.89	4.26	− 0.02	0.00	0.17	78.11	5.14	16.75	0	0
Capgemini D6	1.02	0.61	− 0.10	0.00	0.54	40.91	5.08	53.95	0	0.06
Carrefour	0.94	2.26	− 61.42	− 1.08	0.27	57.06	27.09	15.85	0	0
Carrefour D1	0.91	3.31	0.02	0.00	0.31	62.68	21.65	15.67	0	0
Carrefour D6	0.86	4.77	0.13	0.00	0.59	53.05	9.99	28.83	5.74	2.39
Total	0.96	2.21	− 73.34	− 1.04	0.10	58.49	30.56	10.94	0	0
Total D1	0.96	2.21	− 0.01	0.00	0.10	55.32	33.13	11.54	0	0
Total D6	0.96	2.74	− 0.08	0.00	0.22	53.23	20.81	25.96	0	0
*c) For stocks with an OLS beta greater than 1*										
Saint Gobain	1.12	5.48	− 62.52	− 0.90	0.09	0.12	34.45	65.43	0	0
Saint Gobain D1	1.10	4.54	0.00	0.00	0.11	20.57	34.75	44.68	0	0
Saint Gobain D6	1.02	0.98	0.01	0.00	0.36	26.79	9.45	63.76	0	0
Schneider	1.15	7.38	− 62.12	− 0.86	0.09	0.18	17.70	82.12	0	0
Schneider D1	1.16	7.88	0.02	0.00	0.10	0.24	24.46	75.30	0	0
Schneider D1	1.19	9.05	− 0.10	0.00	0.24	18.60	9.75	71.47	0.06	0.12
Renault	1.16	5.05	− 51.09	− 0.87	0.15	11.36	17.76	70.87	0	0
Renault D1	1.03	1.12	0.03	0.00	0.16	35.47	27.03	37.50	0	0
Renault D6	1.49	14.89	− 0.01	0.00	0.56	23.80	7.72	66.99	0.30	1.20
AXA	1.18	9.45	− 60.95	− 0.82	0.24	27.87	13.04	59.09	0	0
AXA D1	1.12	6.34	0.04	0.00	0.25	28.47	16.03	55.50	0	0
AXA D6	1.19	8.65	− 0.09	0.00	0.43	29.25	12.98	57.60	0.06	0.12
STMI	1.20	5.02	− 39.98	− 0.77	0.36	9.45	35.29	55.26	0	0
STMI D1	1.17	4.44	0.02	0.00	0.37	13.28	37.92	48.80	0	0
STMI D6	0.99	0.19	0.08	0.00	0.74	35.47	5.26	47.43	10.59	1.26
Stellantis	1.21	4.97	− 40.06	− 0.79	0.15	2.63	11.66	85.71	0	0
Stellantis D1	1.07	1.85	0.02	0.00	0.19	18.00	23.56	58.43	0	0
Stellantis D1	1.55	11.49	0.06	0.00	0.89	19.68	3.41	73.15	3.53	0.24
Crédit Agricole	1.31	10.79	− 47.67	− 0.72	0.22	12.56	7.24	80.20	0	0
Crédit Agricole D1	1.24	8.70	0.00	0.00	0.23	15.37	8.13	76.50	0	0
Crédit Agricole D1	1.50	15.66	− 0.06	0.00	0.39	4.43	4.31	91.27	0	0
BNP	1.31	14.50	− 55.96	− 0.75	0.22	4.19	10.47	85.35	0	0
BNP D1	1.28	13.42	0.01	0.00	0.21	9.27	5.68	85.05	0	0
BNP D6	1.24	10.77	− 0.02	0.00	0.35	11.30	8.31	80.38	0	0
Société Générale	1.47	17.25	− 47.81	− 0.67	0.33	12.08	1.61	86.30	0	0
Société Générale D1	1.41	15.74	0.02	0.00	0.36	13.46	7.06	79.49	0	0
Société Générale D6	1.49	15.08	− 0.06	0.00	0.61	12.92	6.04	81.04	0	0

To analyze the frequency robustness of the initial risk-profile, we compare the betas on D1 and D6 to the standard CAPM betas. We find that the values of the short-run and long-run betas can differ from the beta of the standard CAPM. The frequency differentiation of systematic risk (beta values) is consistent with the results of Gençay et al. (2005), Mestre and Terraza (2019), Alexandridis and Hassan (2020), and Mestre (2021). However, beta frequency differentiation does not always imply a risk-profile that is not robust in the frequency domain. Indeed, stocks with an OLS beta of less than 1, indicating a defensive risk-profile, retain this feature in the short and long-term. Only Kering and Capgemini stocks have long-run betas that are significantly equal to or greater than 1, resulting in a different risk-profile in the long-term.

The aggressive stocks (having a CAPM’s beta greater than 1), particularly the financial stocks (Crédit Agricole, BNP, Société Générale, AXA) and Schneider maintain the same feature in the short and long-term. Others’ risk profiles are altered by beta frequency differentiation, as example Stmicroelectronics (STMI) stock with long-term beta significantly equal to 1. The long- and short-run betas for the two stocks with a CAPM beta of 1 (Airbus and LVMH) differ, so their risk-profile changes.

The previous results are consistent with the literature. To improve the risk analysis, we include the time dynamics of the frequency risk-profile in this paper. The results obtained from the ${\beta }_{r}$ serve as a time benchmark, defining the robustness of the initial risk-profile over time.

Regardless of the initial risk-profile, we make the following observations and draw the following remarks. Long-term ${\beta }_{TF}$ are more volatile than short-term ones. This result suggests that the underlying mechanism of systematic risk differs fundamentally in the long-run. Furthermore, the short-term volatility of ${\beta }_{TF}$ is similar to the volatility of ${\beta }_{r}$. The time-varying systematic dynamics of standard rolling beta estimations and wavelets decomposition are relatively similar. Then, we assume that CAPM standards and time-varying parameters implicitly suppose a short-run investment horizon. For some stocks, the high volatility of long-term beta implies a non-robust risk-profile, as their initial characteristics change from time to time. Some stocks’ risk profiles may change more frequently in the long-term while remaining stable in the short-term. Then, investors should monitor this fact when building a long-term portfolio. For example, Hermes stock has a standard CAPM beta lower than 1 and is classified as a defensive stock because 99.46% of the ${\beta }_{r}$ are lower than 1, whereas only 0.54% are equal to 1. Therefore, the risk-profile is robust over time. Its defensive risk-profile is also robust in frequency because its short and long-term betas are both less than 1. However, in the long-run, ${\beta }_{TF}\mathrm{ is}$ more volatile (standard deviation equal to 0.56). Only 58.07% are lower than 1, whereas 17.70% are greater than 1, and 10.23% are lower than 0. Therefore, we suppose that the long-term risk-profile will be unstable over time as many changes occur. The benchmark and the short-term risk-profile have similar characteristics. Similar observations and conclusions can be drawn for other stocks.

We make the following observations after interpreting the results in light of the initial risk-profile:

For stocks with a defensive or low-risk profile (beta < 1), the short-run volatility of ${\beta }_{TF}$ is relatively low (or medium compared with long-run); hence, the risk-profile is robust. However, long-run ${\beta }_{TF}$ is more volatile, and increasing volatility affects the robustness of the risk-profile. We note negative betas, for example, in Dassault, Teleperformance, and Hermes. In this case, a graphical monitoring of the time–frequency-varying betas shows when the change occurs. For stocks with a beta equal or closed to one, the fluctuation of the parameters around 1 indicates a non-robust risk-profile in both the short and long-run. The closer the standard beta is to 1, the less robust the risk-profile. Total stock is an exception because of the low volatility of its short- and long-run betas, which can be interpreted as relatively robust. Note that investors can use their own criteria to determine whether the risk-profile is robust or not. The volatility of ${\beta }_{TF}$ is higher in the long-run than in the short-run for stocks with an aggressive or high-risk profile (beta > 1). We also observe that the closer the beta is to 1, the less robust the risk-profile. However, due to large differences in beta volatility, we cannot determine whether the risk-profile is robust. We note that banking stocks (BNP, SG, and CA) have highly volatile betas, particularly in the long-run, but the risk-profile can be assumed to be robust because 80%–85% of rolling betas are greater than 1.

**Fig. 1:**
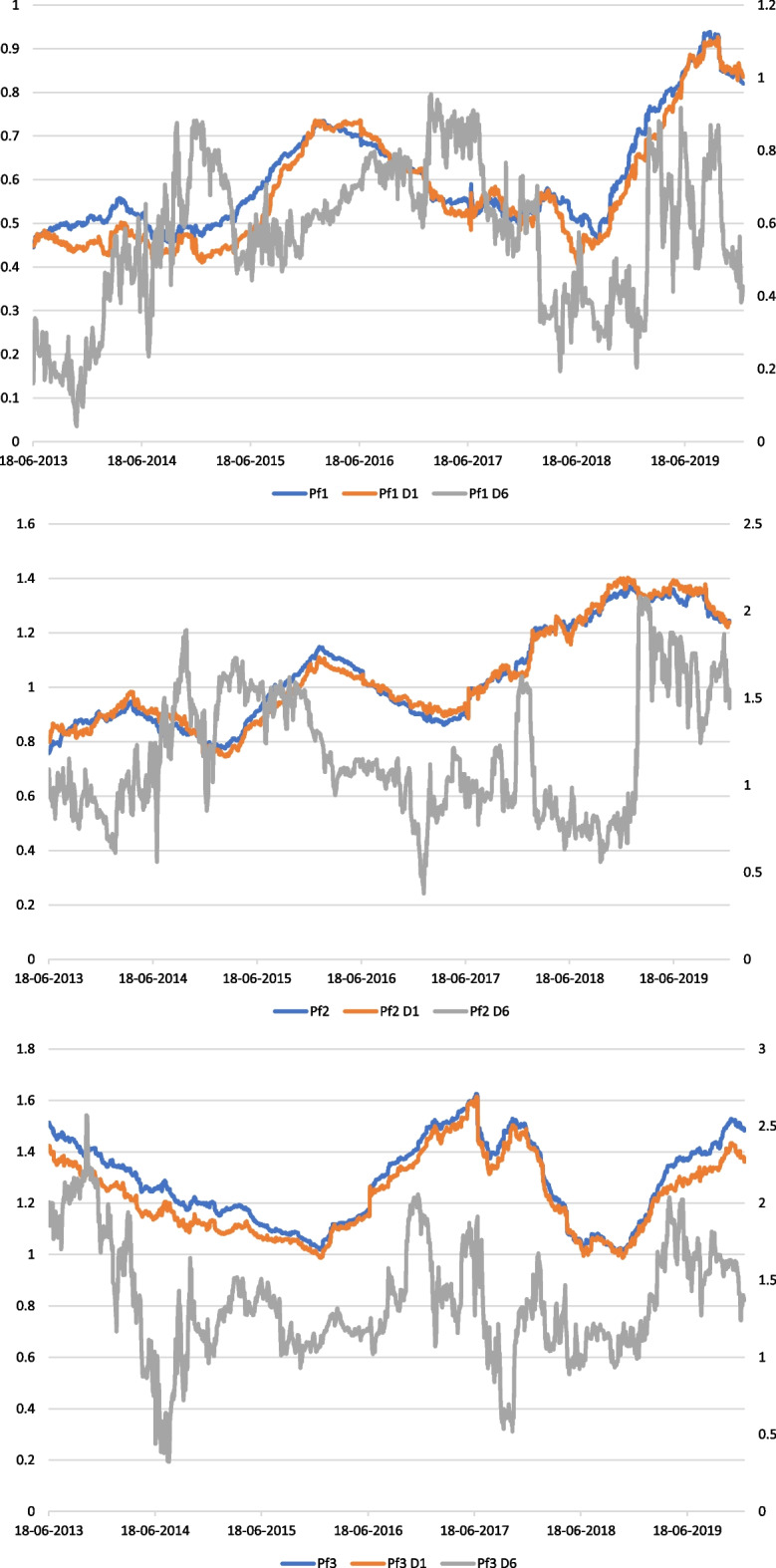
Time–frequency-varying betas of portfolios

To demonstrate the utility of our approach in terms of short- and long-run risk monitoring, we build three portfolios with distinct characteristics. We build a defensive portfolio using five stocks with the lowest beta based on the static value of the CAPM beta. In contrast, we build an aggressive portfolio by selecting five stocks with the highest beta. The two stocks with betas equal to 1 are used to construct a portfolio that tracks market movements. Portfolio 1 (Pf1) is composed of Hermes, Ricard, Dassault, Thales, and Publicis, whereas Portfolio 2 (Pf2) of Airbus and LVMH. Lastly, Portfolio 3 (Pf3) is composed of Crédit Agricole, BNP, Société Générale, Stellantis, and STMI. We used the same wavelet time–frequency window and methods to characterize rolling beta volatility. Table 2 displays the results, and Fig. 1 depicts the rolling betas graphically.

**Table 2:** Portfolios time–frequency risk characteristics

2012–2019	OLS beta	Tstat1	Sharpe	Treynor	SD	%Beta < 1	%Beta = 1	%Beta > 1	%Beta < 0	%Beta = 0
Pf1	0.60	30.86	− 108.10	− 1.61	0.12	100	0	0	0	0
Pf1 D1	0.59	32.41	0.00	0.00	0.13	100	0	0	0	0
Pf1 D6	0.56	36.46	− 0.04	0.00	0.20	99.88	0	0	0	0.12
Pf2	0.99	0.70	− 67.62	− 0.96	0.18	43.48	16.51	40.01	0	0
Pf2 D1	0.99	0.64	0.01	0.00	0.19	43.78	20.63	35.59	0	0
Pf2 D6	1.15	7.49	− 0.04	0.00	0.35	34.45	11.84	53.71	0	0
Pf3	1.30	15.84	− 57.15	− 0.74	0.16	0	9.57	90.43	0	0
Pf3 D1	1.24	13.00	0.02	0.00	0.15	0	16.87	83.13	0	0
Pf3 D6	1.36	15.08	0.00	0.00	0.37	9.09	7.54	83.37	0	0

As expected, we note similarities with Table 1: the long-run volatility of the ${\beta }_{TF}$ is greater than the short-run volatility. The previous one is very closed the volatility of the benchmark ${\beta }_{r}$. Figure 1 depicts the differentiation of the time dynamics of the short-term and long-run betas. The beta values fluctuate in the long-run while being more “smooth” in the short-run. Portfolio 3 results emphasize this point. In the long-run, the standard deviation of ${\beta }_{TF}$ is 0.37, and the betas are in the [0.30–2.6] range. Therefore, 9.09% of the betas are lower than 1. Figure 1 shows, for example, that the initial aggressive risk-profile is not observed in summer 2014 and autumn 2017. However, the beta values are less volatile in the short-run, with 83.13% of betas greater than 1, and the remaining 16.87% equal to 1. The risk-profile is then more robust in the short-run. Portfolio 2 depicts the effects of beta frequency differentiation and volatility on risk-profile robustness. The long-run beta is greater than 1, despite the portfolio being designed to be a tracker. As previously indicated, both Airbus and LVMH have long-run betas greater than 1; thus, the portfolio’s initial feature is lost. Figure 1 shows that the long-run properties of Portfolio 2 are not robust, as betas are less than 1 in 2018 and greater than 1 in 2019. Because 100% of betas are less than 1, Portfolio 1 risk-profile is assumed to be robust in both the short and long-run.

## Time–frequency-varying systematic risk during turmoil and crisis times

We used our methodology in the previous section during a relatively quiet and expanding period. In this section, we assess the robustness of the portfolio risk profiles during turbulent and crisis periods, namely, COVID-19 and the start of the Ukrainian–Russian conflicts.

**Fig. 2:**
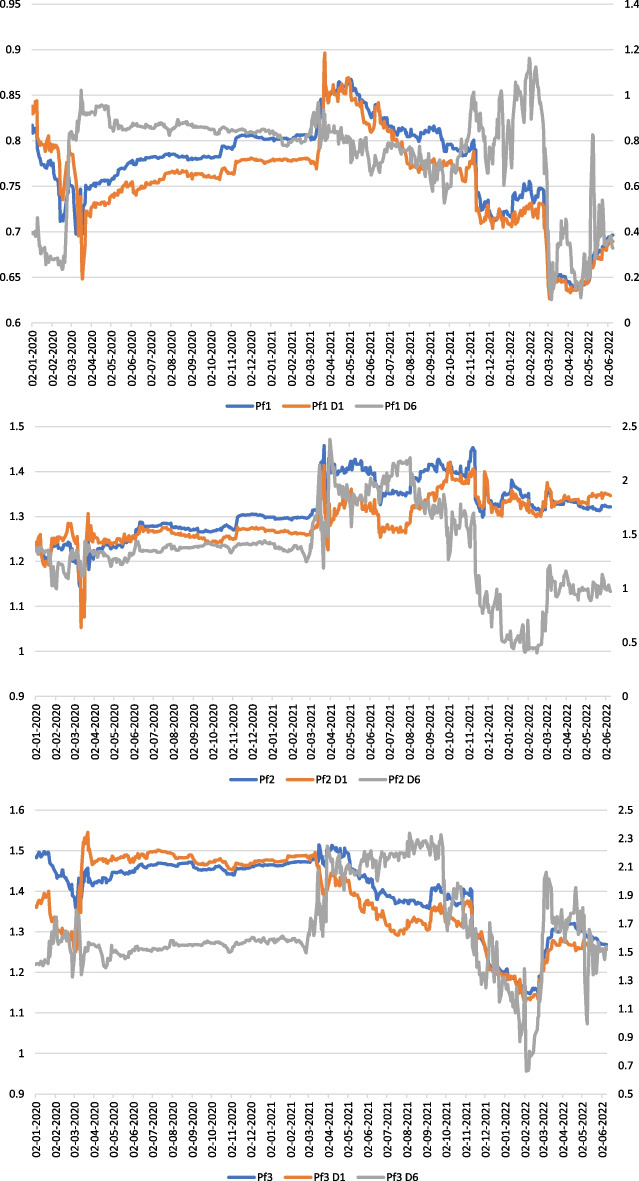
Time–frequency-varying betas of portfolios in the crisis period

We reiterate previous computations for daily data from January 2020 to June 2022. Table 3 and Fig. 2 present our results for the three portfolios we created. Appendix 2 presents the results for all stocks (due to numerous results, graphics are available upon request).

**Table 3:** Portfolios time–frequency risk characteristics in the crisis period

2020–2022	OLS beta	Tstat1	Sharpe	Treynor	SD	%Beta < 1	%Beta = 1	%Beta > 1	%Beta < 0	%Beta = 0
Pf1	0.77	13.59	− 2.30	− 0.04	0.05	100	0	0	0	0
Pf1 D1	0.75	15.36	0.13	0.00	0.05	100	0	0	0	0
Pf1 D6	0.84	8.16	− 0.06	0.00	0.22	94.43	3.98	1.59	0	0
Pf2	1.30	10.98	− 0.46	− 0.01	0.06	0	0	100	0	0
Pf2 D1	1.29	11.70	0.07	0.00	0.05	0	0.16	99.84	0	0
Pf2 D6	1.43	19.17	− 0.04	0.00	0.40	14.01	7.48	78.50	0	0
Pf3	1.40	15.60	− 1.37	− 0.02	0.09	0	0	100	0	0
Pf3 D1	1.39	15.81	0.08	0.00	0.11	0	0	100	0	0
Pf3 D6	1.59	23.93	− 0.12	0.00	0.32	2.55	1.11	96.34	0	0

We note many differences when comparing Tables 1 and 2 because many stocks changed risk profiles during the crisis. Furthermore, because the difference between its short- and long-run betas is significant, more stocks have a mixed risk-profile. As previously stated, we select stocks to construct various types of portfolios with mixed time–frequency features. For example, during a crisis, we build a portfolio with short-run betas less than or close to 1 to attenuate shocks in the short-run, but we construct a portfolio with long-run betas greater than 1 to capitalize on the first signs of recovery in the long-run. We choose Capgemini, Publicis, or Axa (see Appendix 2) to create a portfolio with a short-run beta of 0.93 and a long-run beta of 1.46.

According to standard CAPM, the beta value of Portfolio 2 during this crisis period is greater than 1. The risk-profile established during the expansion period does not hold. It is worth noting that the long-run beta estimated in Table 2 also indicated a beta greater than 1. Hence, if we built Portfolio 2 during the expansion period without considering the long-run beta, we would have underestimated the systematic risk. Airbus is the main source of risk in this portfolio, with betas of 2 in the long-run and 1.50 in the short-term. During the COVID-19 crisis, the tracker portfolio (by design) is actually riskier than the market-like portfolio 3. However, Portfolio 3 is, by construction, riskier because we only included stocks with betas greater than 1. Both short- and long-run betas in Portfolio 3 showed no change in its risk-profile in the frequency domain. The same conclusions can be drawn for Portfolio 1, whose defensive profile is preserved in the frequency domain. As a result, changes to Portfolio 2 are required to conform to the initial tracker characteristic. As an example, we exclude Airbus and include Veolia, which has a short-run beta of 0.89. Because the short-term beta of LVMH is 1.09, the adjusted Portfolio 2 has a short-term beta of 0.99. Michelin can also be included in portfolio 2. In the long-run, LVMH betas are 0.81, and Veolia is 1.21, which is the inverse of the situation in the short-run. The readjusted Portfolio 2 has a beta value of 1. Other stock combinations are possible, with different characteristics, such as a tracker in the short-run but an aggressive in the long-run. Note that we are currently only concerned with static beta in the short and long-run. The following is an example of dynamic adjustments.

In terms of the time dynamics of systematic risk, we observe short and long-run differences in beta volatility. We continue to observe that long-run dynamics are highly erratic compared to short-run dynamics. The short-run risk profiles of portfolios 1 and 3 are robust, as 100% of the rolling betas are lower and greater than 1 across time, respectively. Their respective long-run risk profiles are also considered stable over time. Portfolio 2 and Portfolio 3 have the same profile during this period. Given the revision of Pf2 with Veolia, the long and short-run betas are closer to 1 (see Appendix 3). We propose a potential adjustment, but agents are free to choose other stocks to optimize their portfolio in the long or short-run for a more stable beta over time.

Figure 2 shows a flattening rolling betas curve for the three portfolios from May 2020 to March 2021 due to financial measures and lockdowns during the COVID-19. As betas values vary around their respective static estimations, both short and long-run volatilities are low. Portfolio risk profiles matched their initial features perfectly during this time period. However, the short and long-run risk dynamics become more volatile after the first year of a pandemic.

Long-run rolling betas for Portfolio 1 begin to rise sharply in February 2020. This period corresponded to the Covid-19 outbreak and China’s first quarantine of Hubei province (the most affected areas). The betas reach 1 at the end of March and gradually fall below 1 after the European lockdown. Portfolio 3’s long and short-run betas have been reacting to the Chinese situation since the end of January (Wuhan quarantine). However, short-run betas fall while long-run betas rise. This fact implies that risk perceptions differed early in the pandemic.

The market returned to the post-crisis level around March 2021 (in Europe), and beta volatility became more significant after that date. We also note a break in the beta series around September 2021, which corresponds to the end of the recovery period and the emergence of inflationary pressures and price tensions on energy and raw materials. From September 2021 to February 2022 (market crash), both short and long-run betas for Pf3 fall below 1. During this time, the market exhibits a “plateau effect,” with financial stocks reaching high prices (near highest prices since 2017). The Ukrainian conflict caused a significant increase in betas, followed by a highly erratic fluctuation. Similar conclusions can be drawn for Pf2. However, after the start of the Ukraine war, long-run betas are equal to 1, and the tracker characteristic reappears. However, betas are still greater than 1 in the short-run. Pf1 betas rise from September 2021 to February 2022, reaching greater than 1 just after the Ukrainian conflict and then falling below 1. The “defensive” characteristic is confirmed when the market is stressed, whereas aggressive stocks continue to rise.

## Conclusions

In this paper, we perform forward regression on a time–frequency rolling window to estimate a dynamic beta across time and frequency. We apply this strategy to French stocks and create three portfolios with varying risk profiles. Based on systemic risk dynamics, we propose a method for quantifying the volatility of betas to perform stock profiling monitoring. When the short and long-run betas share the same features (lesser, greater, or equal to 1), the risk-profile is frequency-robust; otherwise, it is not. Furthermore, a risk-profile is time robust when a large part of its rolling betas shares the same features.

The risk profiles of stocks with high beta (high-risk) and low beta (low-risk) tend to be frequency-robust, but the closer the beta to 1, the less frequency-robust the risk-profile is. However, we can identify stocks with short-run betas that differ from long-run betas. The portfolio can then be allocated using a mixed-frequency risk-profile. This result is more frequent during a crisis period. We should also mention that characteristic (aggressive, defensive, or tracker) depends on investment horizons. Our results support the literature by highlighting the frequency differentiation of betas and systematic risks. We should note that a portfolio’s and stocks’ long-run risk-profile can differ from the short-run risk-profile. Agents are free to tailor their portfolio to their preferences.

However, we extend this result because the time–frequency dynamics of risk are also different. Despite high beta volatility, the risk-profile of stocks with high beta is time robust in the long-run, particularly the financial stocks. However, we note a higher proportion of betas equal to or less than 0 for low beta stocks. During a crisis, beta volatility increases, causing drastic changes in the risk-profile of stocks and portfolios. We also notice that the short-run beta dynamics are similar to traditional rolling betas, whereas the long-run dynamics are completely different. The results show that the standard CAPM implicitly assumes short-run investment horizons for both risk (static betas) and time–frequency dynamics. Our application to portfolios also supports this conclusion: long-run investors should consider the time–frequency CAPM rather than the standard CAPM to assess their systematic risk. We confirm Alexandridis and Hassan’s (2020) results that lower frequency beta is more important than higher frequency beta in times of crisis.

Consequently, portfolio risk monitoring should consider investment horizons and the time–frequency dynamics of risk. The differences in short-run and long-run beta dynamics highlight the impact of agents’ perceptions on their systematic risk level. Moreover, the different reactions of short- and long-run betas to the early stages of COVID-19 and the Ukrainian conflict suggest that agents perceive these events differently depending on their investment horizon.

However, we observe that the longer an event lasts, the more the short and long betas tend to follow the same dynamics. We observe a flattening of the betas curve between 2020 and 2021, but the dynamics of short- and long-run betas differ after 2021. These results contradict those of Jain (2022) and Alexandridis and Hasan (2020), who found an increase in beta during the crisis period. Our results are consistent with those of Cao et al. (2022), who found that stock betas tend to decrease as the pandemic severity increases. According to Abd-Alla (2020) and Jain (2020), capitalization and firm sector tend to modify the effect of the crisis on beta coefficients. On this point, we partially confirm their results because we note different evolutions of the beta parameters depending on the equities. Concerning the effects of the Ukrainian conflict, our results are consistent with the literature, which indicates an increasing beta in times of crisis. Finally, the effects of COVID-19 and the Ukrainian conflict on equity betas are related to investment horizons and initial risk-profile. We can conclude that the COVID-19 period impacts the systematic risk differently than the Ukrainian Conflict.

In sum, the methodology developed in this paper can be tailored to the users’ needs by varying the window length or by performing a recursive regression backward or forward. Furthermore, this wavelet approach can be associated with other clustering algorithms, such as a multiple criteria decision-making model, to automate the risk-profile or stock selection, as demonstrated by Li et al. (2021) and Kou et al. (2014). This methodology is then suitable to easily access the time–frequency volatility of the systematic risk, providing a powerful tool for better tailing and monitoring portfolio characteristics at each new price discovered and according to its investment horizon.

## Data Availability

The datasets analysed during the current study are freely available in Yahoo Finance repository.

## Appendix

### *Appendix 1* MODWT Frequency Bands and related investment horizons in days

**Table:**

Frequency Bands	Inf Border	Sup Border
D1	2	4
D2	4	8
D3	8	16
D4	16	32
D5	32	64
D6	64	128
D7	128	256
D8	256	512
D9	512	1024
D10	1024	2048
D11	2048	4096

### *Appendixes 2* Stocks time-frequencies risk characteristics in turmoil period

- For stock with an OLS Beta equal to 1

**Table:**

2020–2022	OLS beta	Tstat1	Sharpe	Treynor	SD	%Beta < 1	%Beta = 1	%Beta > 1	%Beta < 0	%Beta = 0
Veolia	0.94	1.79	− 1.32	− 0.03	0.16	74.20	21.50	4.30	0	0
Veolia D1	0.89	3.29	0.00	0.00	0.16	90.45	5.73	3.82	0	0
Veolia D6	1.21	7.01	− 0.15	0.00	0.38	36.62	6.53	56.69	0	0.16
Michelin	0.98	0.68	− 1.72	− 0.03	0.11	40.92	52.55	6.53	0	0
Michelin D1	0.92	2.51	0.08	0.00	0.10	60.35	39.65	0	0	0
Michelin D6	1.01	0.46	− 0.06	0.00	0.46	57.96	20.70	21.34	0	0
Capgemini	0.99	0.18	0.32	0.01	0.04	32.48	64.81	2.71	0	0
Capgemini D1	0.92	2.32	0.04	0.00	0.10	65.29	23.09	11.62	0	0
Capgemini D6	1.52	18.82	− 0.11	0.00	0.28	4.14	5.57	90.29	0	0
Publicis	1.00	0.00	− 2.60	− 0.06	0.15	37.58	21.02	41.40	0	0
Publicis D1	0.87	3.07	0.05	0.00	0.16	71.02	17.99	10.99	0	0
Publicis D6	1.20	7.46	− 0.08	0.00	0.55	22.93	3.34	64.65	6.37	2.71

b) For stock with an OLS Beta lesser than 1

**Table:**

2020–2022	OLS beta	Tstat1	Sharpe	Treynor	SD	%Beta < 1	%Beta = 1	%Beta > 1	%Beta < 0	%Beta = 0
Sanofi	0.49	18.37	− 2.85	− 0.08	0.10	100	0	0	0	0
Sanofi D1	0.50	19.59	− 0.05	0.00	0.10	100	0	0	0	0
Sanofi D6	0.54	22.49	− 0.11	0.00	0.34	86.78	2.07	8.12	0.96	2.07
Eurofins	0.49	9.99	1.73	0.08	0.29	86.62	0.80	2.87	0.16	9.55
Eurofins D1	0.59	8.27	0.09	0.00	0.33	82.32	2.39	3.66	0.16	11.46
Eurofins D6	0.31	14.65	− 0.18	0.00	0.65	61.94	0.16	3.50	31.53	2.87
Carrefour	0.51	12.99	− 2.42	− 0.08	0.14	100	0	0	0	0
Carrefour D1	0.53	12.73	0.01	0.00	0.16	100	0	0	0	0
Carrefour D6	0.34	21.14	− 0.18	0.00	0.37	74.04	3.66	6.37	10.51	5.41
Orange	0.51	17.98	− 7.66	− 0.20	0.17	100	0	0	0	0
Orange D1	0.53	17.76	− 0.08	0.00	0.19	100	0	0	0	0
Orange D6	0.37	22.16	− 0.22	0.00	0.39	75.96	6.53	14.49	0.32	2.71
Danone	0.55	15.60	− 6.18	− 0.16	0.08	100	0	0	0	0
Danone D1	0.59	14.95	0.16	0.00	0.06	100	0	0	0	0
Danone D6	0.65	14.52	− 0.22	0.00	0.27	85.19	4.46	4.14	6.05	0.16
Vivendi	0.62	8.55	− 3.22	− 0.11	0.16	100	0	0	0	0
Vivendi D1	0.63	8.32	0.03	0.00	0.24	100	0	0	0	0
Vivendi D6	0.72	6.94	− 0.38	0.00	0.67	61.62	2.71	29.14	5.89	0.64
Ricard	0.63	13.99	− 3.51	− 0.08	0.06	100	0	0	0	0
Ricard D1	0.62	14.39	0.14	0.00	0.10	100	0	0	0	0
Ricard D6	0.60	16.92	0.00	0.00	0.24	94.59	0.32	0	1.91	3.18
Dassault	0.68	8.44	− 0.07	0.00	0.15	96.18	3.03	0.80	0	0
Dassault D1	0.74	6.75	0.15	0.00	0.17	92.36	1.75	5.89	0	0
Dassault D6	0.78	5.51	− 0.09	0.00	0.64	61.15	3.03	17.83	11.46	6.53
Téléperformance	0.73	6.63	0.81	0.02	0.21	97.45	2.55	0	0	0
Téléperformance D1	0.78	5.18	0.09	0.00	0.26	90.45	9.24	0.32	0	0
Téléperformance D6	0.89	3.19	− 0.07	0.00	0.52	46.97	24.04	7.64	15.29	6.05
Thales	0.74	6.39	− 3.06	− 0.08	0.33	50.80	23.89	23.09	0	2.23
Thales D1	0.67	8.80	0.03	0.00	0.30	69.59	12.42	10.35	0	7.64
Thales D6	0.98	0.48	− 0.03	0.00	0.81	47.13	7.80	30.10	13.22	1.75
Air liquide	0.75	12.07	− 1.33	− 0.02	0.10	99.84	0.16	0	0	0
Air liquide D1	0.83	8.24	0.09	0.00	0.17	82.01	17.04	0.96	0	0
Air liquide D6	0.65	18.35	− 0.09	0.00	0.19	88.69	5.25	6.05	0	0
L’oreal	0.77	8.63	− 1.19	− 0.02	0.08	98.09	1.91	0	0	0
L’oreal D1	0.80	7.57	0.20	0.00	0.10	89.97	10.03	0	0	0
L’oreal D6	0.53	18.19	− 0.17	0.00	0.44	73.57	5.41	21.02	0	0
Hermès	0.81	6.62	1.31	0.03	0.13	86.94	12.74	0.32	0	0
Hermès D1	0.86	4.84	0.10	0.00	0.12	85.67	6.05	8.28	0	0
Hermès D6	0.61	10.38	0.00	0.00	0.56	65.76	3.50	14.81	12.74	3.18
Alstom	0.84	3.58	− 4.50	− 0.12	0.13	89.17	3.98	6.85	0	0
Alstom D1	0.89	2.43	0.12	0.00	0.12	89.17	5.41	5.41	0	0
Alstom D6	0.98	0.34	− 0.12	0.00	0.86	45.38	0.96	47.93	2.87	2.87
Legrand	0.88	4.54	− 1.20	− 0.02	0.11	88.85	11.15	0	0	0
Legrand D1	0.88	4.62	0.18	0.00	0.15	70.22	29.30	0.48	0	0
Legrand D6	0.73	10.88	− 0.14	0.00	0.32	86.15	1.91	10.83	0.64	0.48
Essilor	0.88	3.94	− 2.12	− 0.04	0.06	89.49	10.51	0	0	0
Essilor D1	0.85	4.89	0.06	0.00	0.08	90.61	9.08	0.32	0	0
Essilor D6	0.82	7.58	− 0.09	0.00	0.43	79.46	7.17	6.85	6.53	0
Engie	0.91	2.81	− 3.95	− 0.08	0.14	60.51	39.49	0	0	0
Engie D1	0.84	5.46	− 0.01	0.00	0.11	99.68	0.32	0	0	0
Engie D6	1.28	7.01	− 0.13	0.00	0.37	42.99	4.94	52.07	0	0

iii) For stock with an OLS Beta greater than 1

**Table:**

2020–2022	OLS beta	Tstat1	Sharpe	Treynor	SD	%Beta < 1	%Beta = 1	%Beta > 1	%Beta < 0	%Beta = 0
Bouygues	1.07	2.20	− 3.32	− 0.06	0.29	25.80	17.68	56.53	0	0
Bouygues D1	1.10	3.24	− 0.01	0.00	0.32	22.61	20.06	57.32	0	0
Bouygues D6	0.82	6.60	− 0.28	0.00	0.50	63.06	2.71	32.48	0	1.75
LVMH	1.09	3.45	1.30	0.02	0.13	16.08	37.58	46.34	0	0
LVMH D1	1.09	3.70	0.06	0.00	0.13	5.57	40.76	53.66	0	0
LVMH D6	0.81	10.60	− 0.03	0.00	0.26	56.85	6.69	36.46	0	0
Total	1.09	2.65	− 2.14	− 0.04	0.23	29.62	1.59	68.79	0	0
Total D1	1.04	1.31	0.00	0.00	0.20	32.32	4.30	63.38	0	0
Total D6	1.19	4.70	− 0.04	0.00	0.93	13.54	1.75	74.52	9.24	0.96
Schneider	1.09	3.92	0.71	0.01	0.09	18.15	30.73	51.11	0	0
Schneider D1	1.08	3.28	0.12	0.00	0.09	19.43	36.78	43.79	0	0
Schneider D1	0.80	6.95	− 0.19	0.00	0.37	72.13	3.82	24.04	0	0
AXA	1.10	3.74	− 2.47	− 0.04	0.16	31.21	0.96	67.83	0	0
AXA D1	1.00	0.19	0.03	0.00	0.13	52.71	5.57	41.72	0	0
AXA D6	1.66	27.45	− 0.14	0.00	0.61	0	0	100	0	0
Kering	1.11	3.38	− 2.06	− 0.04	0.10	3.03	38.22	58.76	0	0
Kering D1	1.10	3.15	0.06	0.00	0.11	12.42	38.85	48.73	0	0
Kering D6	0.72	8.95	− 0.04	0.00	0.56	67.52	4.46	27.23	0.32	0.48
Saint Gobain	1.20	6.13	0.11	0.00	0.12	4.46	26.59	68.95	0	0
Saint Gobain D1	1.13	4.13	0.12	0.00	0.13	24.36	14.01	61.62	0	0
Saint Gobain D6	1.31	14.29	− 0.15	0.00	0.32	19.43	3.82	76.75	0	0
Vinci	1.23	7.43	− 1.93	− 0.03	0.21	22.45	5.57	71.97	0	0
Vinci D1	1.26	8.22	0.02	0.00	0.25	16.72	8.76	74.52	0	0
Vinci D6	1.28	10.78	− 0.10	0.00	0.49	22.77	2.39	71.66	2.39	0.80
CA	1.31	8.66	− 2.53	− 0.05	0.16	4.46	6.21	89.33	0	0
CA D1	1.28	8.09	0.05	0.00	0.14	3.34	9.39	87.26	0	0
CA D1	1.93	25.35	− 0.08	0.00	0.73	5.57	1.75	92.68	0	0
STMI	1.32	7.22	2.03	0.04	0.23	0	10.19	89.81	0	0
STMI D1	1.32	7.40	0.12	0.00	0.21	0	0	100	0	0
STMI D6	1.22	6.67	− 0.14	0.00	0.66	12.90	5.41	66.24	10.51	4.94
BNP	1.36	9.96	− 1.69	− 0.03	0.15	0	1.27	98.73	0	0
BNP D1	1.34	10.35	0.02	0.00	0.11	0	0.16	99.84	0	0
BNP D6	1.74	17.60	− 0.10	0.00	0.69	10.83	1.75	86.94	0.48	0
Stellantis	1.43	10.80	− 1.31	− 0.02	0.07	0	0	100	0	0
Stellantis D1	1.43	11.14	0.15	0.00	0.10	0	0	100	0	0
Stellantis D6	1.30	11.33	− 0.14	0.00	0.42	0.16	1.59	98.25	0	0
Safran	1.51	11.06	− 2.80	− 0.05	0.22	0	7.48	92.52	0	0
Safran D1	1.48	11.19	0.13	0.00	0.22	2.71	5.73	91.56	0	0
Safran D6	1.86	21.25	0.01	0.00	0.83	19.27	2.87	74.36	2.55	0.96
Airbus	1.52	10.49	− 1.50	− 0.03	0.17	0	0	100	0	0
Airbus D1	1.49	11.05	0.06	0.00	0.11	0	0	100	0	0
Airbus D6	2.04	23.37	− 0.04	0.00	0.92	19.43	1.59	72.93	4.94	1.11
SG	1.60	13.34	− 2.50	− 0.05	0.18	0	0	100	0	0
SG D1	1.59	13.42	0.02	0.00	0.22	0	0	100	0	0
SG D6	1.78	14.21	− 0.10	0.00	0.78	7.80	2.07	89.65	0	0.48
Renault	1.61	11.41	− 4.68	− 0.09	0.23	0	1.59	98.41	0	0
Renault D1	1.65	12.46	0.09	0.00	0.27	0.16	3.66	96.18	0	0
Renault D6	1.83	15.06	− 0.02	0.00	0.79	4.14	3.03	89.01	3.66	0.16

### *Appendixes 3* Short and Long run Rolling Betas of adjusted portfolio 2

The new portfolio 2 (noted NPf2) is adjusted with LVMH and Veolia while original portfolio 2 is composed by LVMH and Airbus.

## Abbreviations

CAPM: Capital assets pricing model
RMT: Random matrix theory
APT: Arbitrage pricing theory
OLS: Ordinary least squares
MODWT: Maximal overlap discrete wavelets transform
CWT: Continuous wavelets transform
ICWT: Inverse continuous wavelets transform
GARCH: Generalised autoregressive conditionnal heteroskedastic
CAC40: Cotations assistées continues 40 (main index of Bourse de Paris)
OAT: Obligation assimilable du Trésor (French treasury bonds rate)
La8: Least asymmetric Daubechies wavelet 8
Tstat: Student statistic
LVMH: Louis vuitton moet hennessy
CA: Crédit agricole
STMI: STMicroelectronics

## Declarations

### Consideration for publication

This manuscript has not been published and is not under consideration for publication elsewhere.
